# Novel approaches for random-effects meta-analysis of a small number of studies under normality

**DOI:** 10.1017/rsm.2025.10022

**Published:** 2025-07-10

**Authors:** Yajie Duan, Thomas Mathew, Demissie Alemayehu, Ge Cheng

**Affiliations:** 1 Department of Statistics, Rutgers University, New Brunswick, NJ, USA; 2 Department of Mathematics and Statistics, University of Maryland, Baltimore County, MD, USA; 3 Statistical Research and Data Science Center, Pfizer Inc., New York, NY, USA

**Keywords:** confidence interval, fiducial inference, modified LRT statistic, rare diseases, small sample asymptotics

## Abstract

Random-effects meta-analyses with only a few studies often face challenges in accurately estimating between-study heterogeneity, leading to biased effect estimates and confidence intervals with poor coverage. This issue is especially the case when dealing with rare diseases. To address this problem for normally distributed outcomes, two new approaches have been proposed to provide confidence limits of the global mean: one based on fiducial inference, and the other involving two modifications of the signed log-likelihood ratio test statistic in order to have improved performance with small numbers of studies. The performance of the proposed methods was evaluated numerically and compared with the Hartung–Knapp–Sidik–Jonkman approach and its modification to handle small numbers of studies. The simulation results indicated that the proposed methods achieved coverage probabilities closer to the nominal level and produced shorter confidence intervals compared to those based on existing methods. Two real examples are used to illustrate the proposed methods.

## Highlights

### What is already known?

- Random-effects meta-analyses with few studies often struggle to accurately estimate between-study heterogeneity, resulting in biased effect estimates and poorly covered confidence intervals. For normally distributed outcomes, the Hartung–Knapp–Sidik–Jonkman (HKSJ) method adjusts the CI for the global mean. Rover et al. proposed a modified Knapp–Hartung (mKH) method to improve performance with a small number of studies.

### What is new?

- We proposed two new approaches to construct confidence intervals for the global mean: one based on fiducial inference and another using modified signed log-likelihood ratio test statistics for improved small-sample performance. Simulations showed that the new methods yielded coverage probabilities closer to nominal levels and shorter intervals than the existing methods.

### Potential impact for RSM readers

- Random-effects meta-analyses with a small number of studies are a common challenge, particularly in areas such as rare disease research. The proposed approaches offer readers effective alternatives with improved performance for meta-analyses with few studies, helping researchers make more informed decisions.

## 1 Introduction

Conducting a meta-analysis often involves a random-effects model that can account for both within-study and between-study variability by using a normal–normal hierarchical model.^1^ For outcomes that follow a normal distribution, the data are represented as *k* estimates 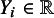 with known within-trial variances 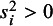 : (1)

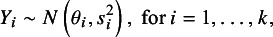

where the 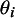 s vary from trial to trial around a global mean 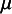 according to the normal distribution (2)

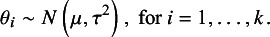

Here, 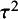 is a heterogeneity variance component, representing the variability between trials or studies. The integrated and simplified model under consideration, as discussed in,^1^ is (3)

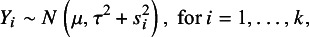

where 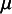 is the unknown parameter of interest. The within-trial variances 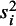 , assumed to be known, depend on the group sizes in each trial and on the within-trial variances of the outcome variables in the *i*th trial. The latter are typically estimated, even though we proceed under the standard assumption that these are known. In many applications, only a limited number of studies is available for a meta-analysis, often fewer than five. Estimating the between-study heterogeneity 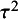 is particularly challenging in these situations. Inaccurate heterogeneity estimates can result in biased effect estimates and overly narrow confidence intervals.^2^ Deriving reliable estimates and confidence intervals from only a small number of studies is challenging, especially in the context of rare diseases.

The problem of interest in our work is the computation of confidence limits for the underlying mean 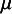 when the number of studies *k* is small. Mathes and Kuss^2^ demonstrated that for binary outcomes and in the presence of random effects, the beta-binomial model was the most appropriate model for meta-analysis involving a small number of studies, offering a balanced trade-off between coverage probability and power. For normally distributed outcomes, there are several methods to construct confidence intervals for 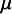 . However, there is no universally accepted satisfactory method, especially for small *k*. In the present work, the focus is on the outcomes following a normal distribution.

A natural point estimate of 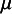 can be obtained as a weighted average of the 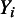 s in (3), weighted by the inverse of the variance in the model (3). The resulting point estimate, say 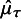 , and the associated normal distribution, are given by (4)

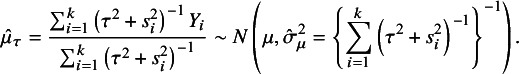

Using a normal approximation for 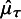 , an approximate 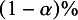 confidence interval for 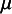 is given by (5)

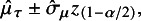

where 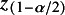 is the 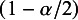 -quantile of the standard normal distribution. In practice, 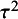 is replaced with a suitable estimate, and the normal approximation is satisfactory when the number of studies *k* is large, with small within-trial variances or when heterogeneity is minimal.^1^

An adjusted CI introduced by Hartung and Knapp,^3^ and Sidik and Jonkman,^4^ usually referred to as the Hartung–Knapp–Sidik–Jonkman (HKSJ) method is given by (6)

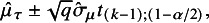

where 
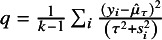
 , and 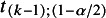 is the 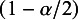 -quantile of the Student-*t* distribution with 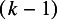 degrees of freedom. For a small number of studies *k*, Rover et al.^1^ proposed the modified Knapp–Hartung (mKH) method, replacing *q* by (7)

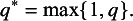

The CI of 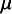 using the HKSJ method in (6) is generally wider than the CI based on the normal approximation in (5) since the student-t quantile is larger than the corresponding normal quantile. However, *q* can become arbitrarily small, and if 
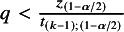
 , the HKSJ method’s adjusted interval would be shorter than the normal approximation-based interval, which is counter-intuitive. The mKH method provides a more conservative approach, with error rates that are more closely aligned with the nominal level, particularly when dealing with a small number of studies with varying sizes or precision levels.^1^

In this work, two novel approaches are proposed to construct confidence intervals for 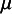 , and their performance is evaluated numerically through a simulation study, and compared with the existing methods. The first approach is based on the idea of fiducial inference, and the second approach is based on two modifications to the signed log-likelihood ratio test statistic to obtain accurate small sample performance.^5^ For details on fiducial inference, we refer to the works of Hannig et al.^6^^,^ ^7^ Earlier, fiducial quantities were investigated by Weerahandi,^8^ referring to it as generalized pivotal quantities.

The following sections first describe the proposed methods and algorithms for obtaining confidence limits of 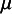 in (3). Next, the performance of these methods is evaluated through the results of a simulation study and two applied examples. The paper concludes with a discussion of the findings.

## 2 Methods

To obtain the confidence limits for 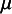 in the model (3), especially when the number of studies *k* is small, two methods are proposed. One method is based on the fiducial approach, while the other involves modifications of the signed log-likelihood ratio test statistic. The fiducial approach is primarily based on the work of Iyer et al.^9^ The second method, which focuses on small sample asymptotics, includes two modifications to the signed log-likelihood ratio test to improve performance in small sample scenarios, as detailed by DiCiccio et al.^5^

### 2.1 The fiducial approach

Consider the point estimate of 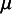 , denoted by 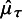 , given in equation (4). Let the corresponding residual sum of squares be denoted by 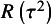 . Defining (8)

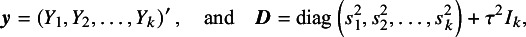

the quantity 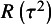 can be expressed as (9)

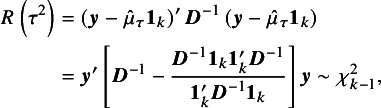

where 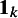 is a 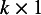 vector of ones, and 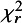 denotes the central chi-square distribution with *r* degrees of freedom. The quantities 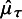 and 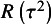 are independent, as shown by the standard results on linear models under the normality assumption.

To understand the behavior of 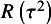 as a function of 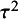 , consider another representation of 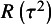 . Let 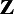 be a 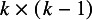 matrix whose columns are orthonormal and orthogonal to the vector 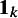 (i.e., 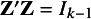 and 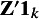 is a vector of zeros). Then, the following result holds: 

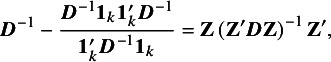

see Appendix M, Section M.4.f in the work by Searle et al.^10^ Thus, 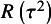 can be represented as (10)

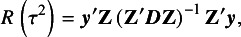

where 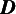 is the diagonal matrix given in Equation (8). From this representation, it follows that 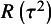 is a decreasing function of 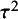 , reaching its maximum value when 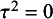 , and is also a convex function of 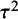 . These properties were also proved by Iyer et al.^9^ based on the representation (9). Let (11)

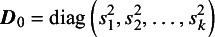

which is the value of 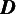 in (8) when 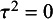 . Thus, 
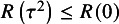
 . Note that (12)

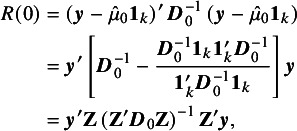

where 
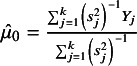
 . We note that 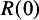 is the usual 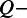 statistic to test if 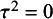 , and 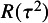 is the generalized 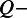 statistic.^11^

We shall now explain the derivation of a fiducial quantity for 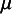 , whose percentiles can be used to compute confidence limits for 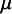 . Since the fiducial quantity for 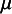 depends on the fiducial quantity for 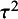 , we shall first obtain a fiducial quantity for the latter. Let 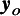 denote the observed value of 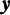 . Furthermore, let (13)

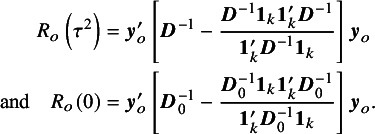

Let 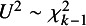 . Following the work of Iyer et al.,^9^ a fiducial quantity for 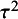 is the solution to the equation 
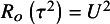
 , provided 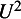 is between 0 and 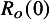 . Thus 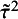 , the fiducial quantity for 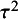 , is given by (14)

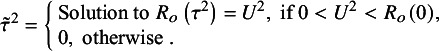

Here, we would like to note that Paule and Mandel^12^ suggested an estimator of 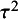 as the solution to 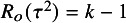 .

To get a fiducial quantity for 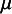 , note that 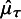 in (4) can be written as 

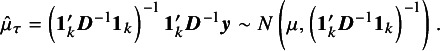

Thus, we can write (15)

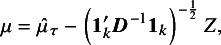

where 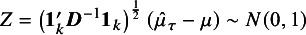 . The expression on the right-hand side of the above equation depends on the unknown parameter 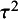 and the observable random variable 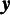 (in the expression for 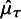 ). A fiducial quantity for 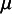 is obtained from the above equation by replacing 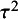 with the fiducial quantity 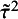 in (14) and by replacing 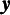 with its observed value 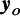 . Thus, let (16)

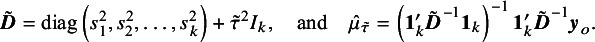

A fiducial quantity for 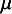 , denoted by 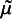 , is then given by (17)

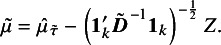



We note that 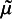 is a function of two independent random variables: the chi-square random variable 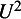 with 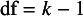 used in the computation of 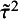 in (14), and a standard normal random variable *Z*. Once the observed data vector 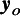 is available, a large number of values, say *m*, of the pair 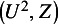 can be generated. These *m* pairs of values, along with the observed data 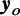 , can be used to compute *m* realizations of the fiducial quantities 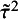 and 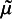 . The percentiles of the *m* realizations of 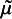 provide confidence limits for 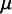 .

In the above approach, point estimates of 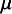 and 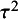 are not explicitly used. An algorithm for implementing the fiducial approach to obtain confidence limits for 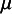 is outlined below.

**Figure:**
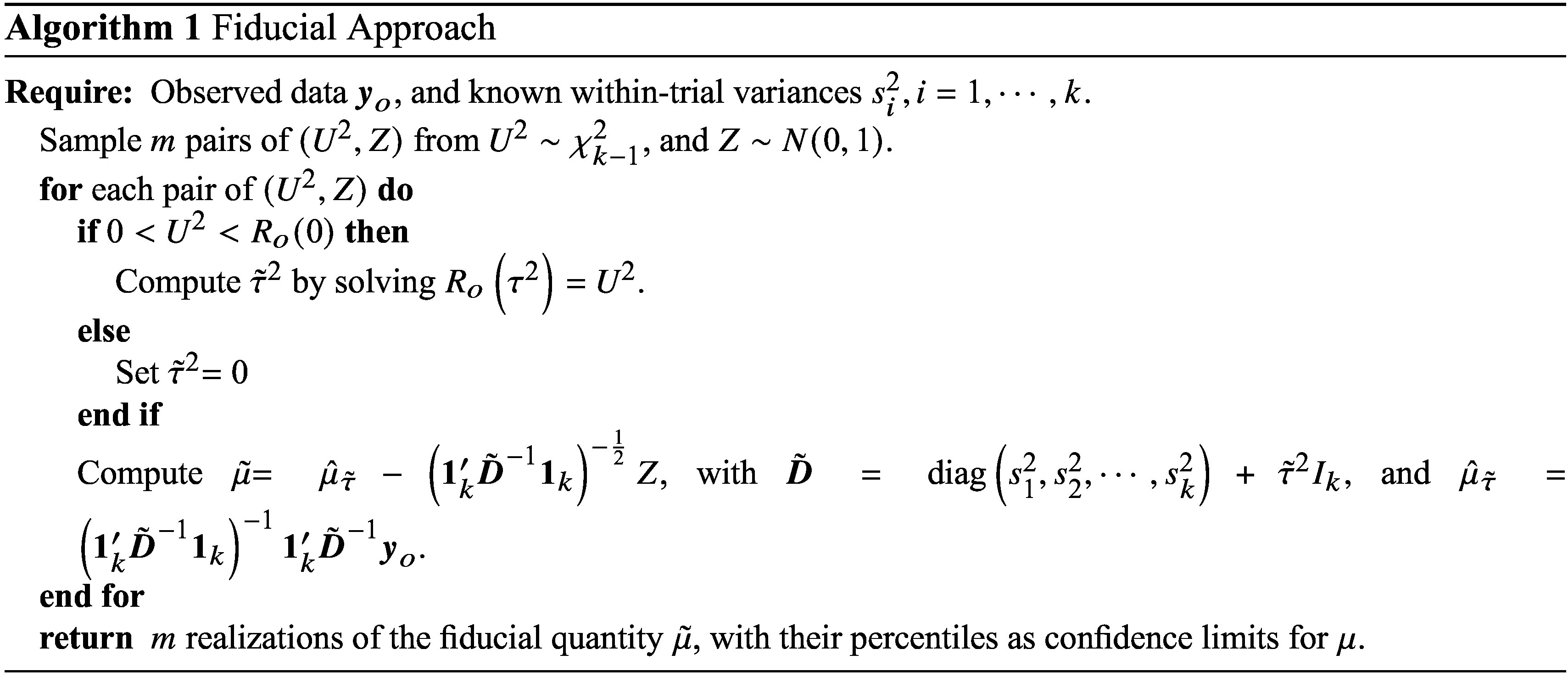

### 2.2 Small sample asymptotics

It is well known that under regularity conditions, the signed log-likelihood ratio test statistic is asymptotically standard normal, providing first-order accuracy. Small-sample asymptotics involve modifying the signed log-likelihood ratio test statistic to achieve accurate performance in small samples. In order to introduce the basic idea, consider an 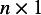 vector of observations 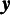 whose distribution depends on a 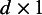 parameter vector 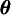 . Let 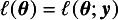 be the log-likelihood function. Suppose that the parameter 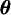 is partitioned as 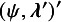 , where 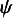 is a scalar parameter of interest, and 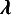 is a nuisance parameter. Let 
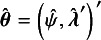
 denote the MLE, 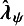 denote the constrained MLE of 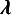 for a fixed 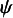 , and write 
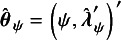
 . Large sample inference for 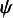 can be based on the signed log-likelihood root (18)

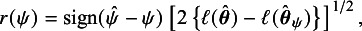

where 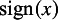 is +1 if 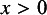 and -1 if 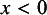 . Under regular conditions, 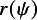 is asymptotically standard normal with an error of the order 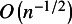 . Several modifications of 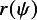 are available in the literature to improve small sample performance.^13^ Here, we consider two modifications proposed by DiCiccio et al.^5^

#### 2.2.1 Modification I

To describe the first modification, let 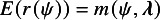 and 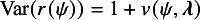 . Now define (19)

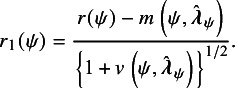

For a fixed value of 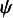 , 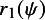 is thus a standardization of 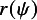 using the mean and variance evaluated at 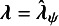 . DiCiccio et al.^5^ have shown that under regular conditions, 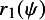 has an asymptotic standard normal distribution, and 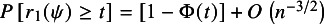 . In other words, the tail area approximation based on the asymptotic standard normal distribution of 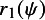 is significantly more accurate than that based on the asymptotic standard normal distribution of 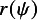 .

In order to carry out inference based on the asymptotic standard normal distribution of 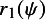 , it is necessary to compute the mean and variance of 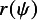 ; namely, the quantities 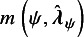 and 
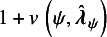
 . For a specified value of 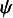 , these can be obtained numerically, as noted in the work of DiCiccio et al.^5^ After specifying 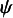 , compute 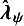 , and generate a parametric bootstrap sample of size *n* when 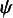 takes the specified value and 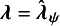 . Let 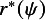 denote the value of 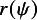 calculated from the parametric bootstrap sample. Generate several parametric bootstrap samples of size *n*, say *B* samples, and compute *B* values of 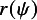 , denoted by 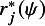 for 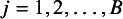 . The mean and variance of 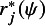 for 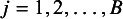 , provide estimates of 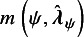 and 
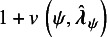
 , respectively, to be used in the calculation of 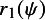 . To compute confidence limits for 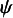 based on 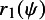 , equate 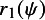 to standard normal percentiles and solve for 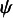 . For example, a 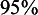 upper confidence limit for 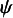 can be obtained as the solution for 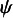 to equation 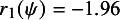 .

#### 2.2.2 Modification II

A second modification suggested by DiCiccio et al.^5^ involves estimating the tail probability 
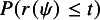
 using Monte Carlo simulation at the parameter value 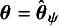 , rather than relying on the standard normal distribution for 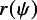 . For example, to test the null hypothesis 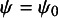 against the one-sided alternative 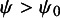 , a *p*-value can be calculated as 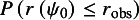 , evaluated at the parameter value 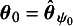 , where 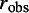 is the observed value of 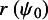 . The confidence limits for 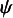 can then be obtained by inverting the test.

#### 2.2.3 Algorithms

In the context of the random-effects meta-analysis model given in (3), the parameter of interest is 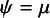 , and the nuisance parameter is the scalar 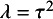 . Thus, in terms of the notation used for the two modifications given above, the parameter vector is 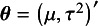 . We use 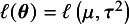 to denote the log-likelihood function under the model (3).

The algorithms proposed to implement the two modifications to obtain the confidence limits of 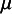 under the model (3) are presented below.

**Figure:**
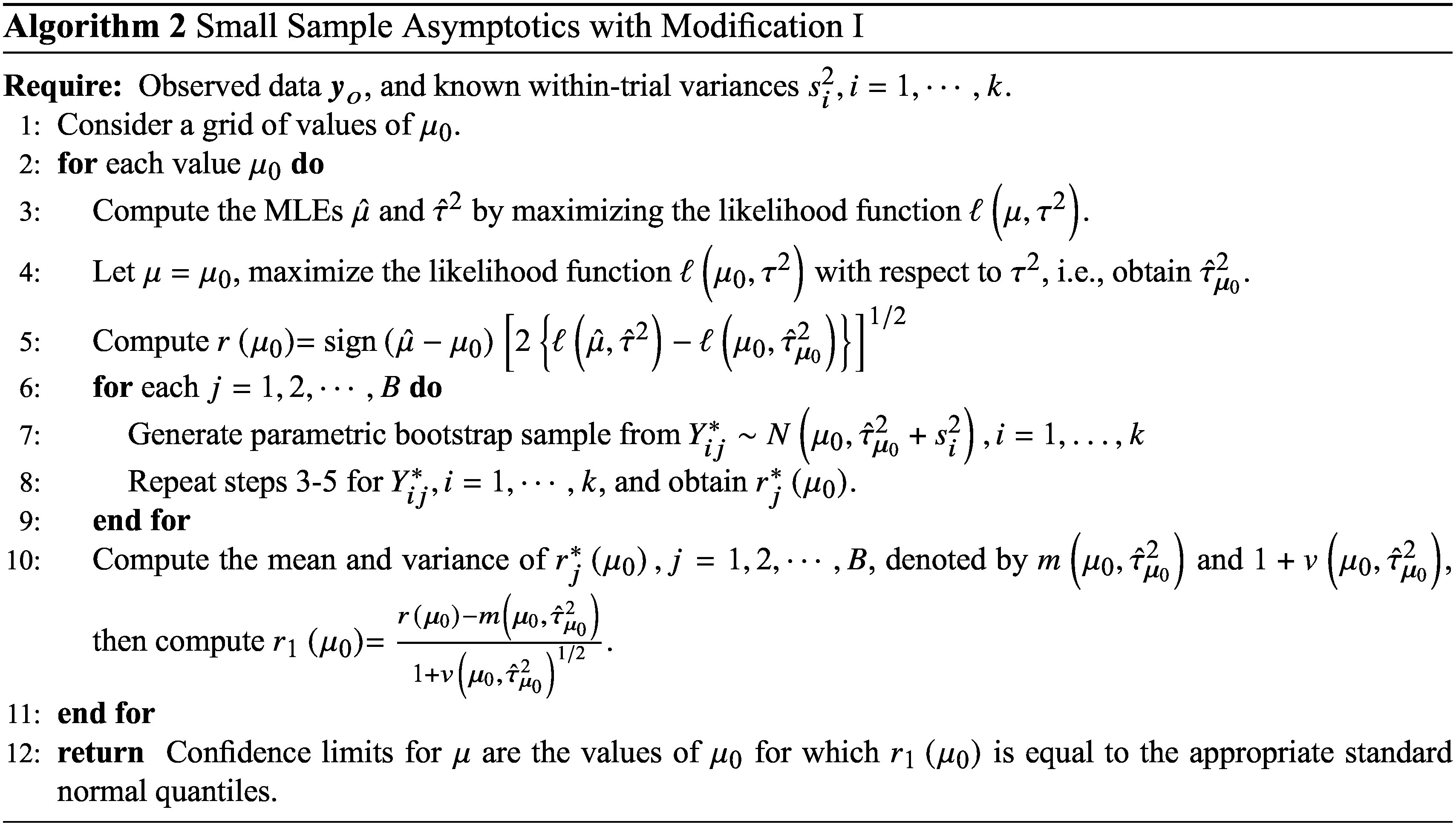

**Figure:**
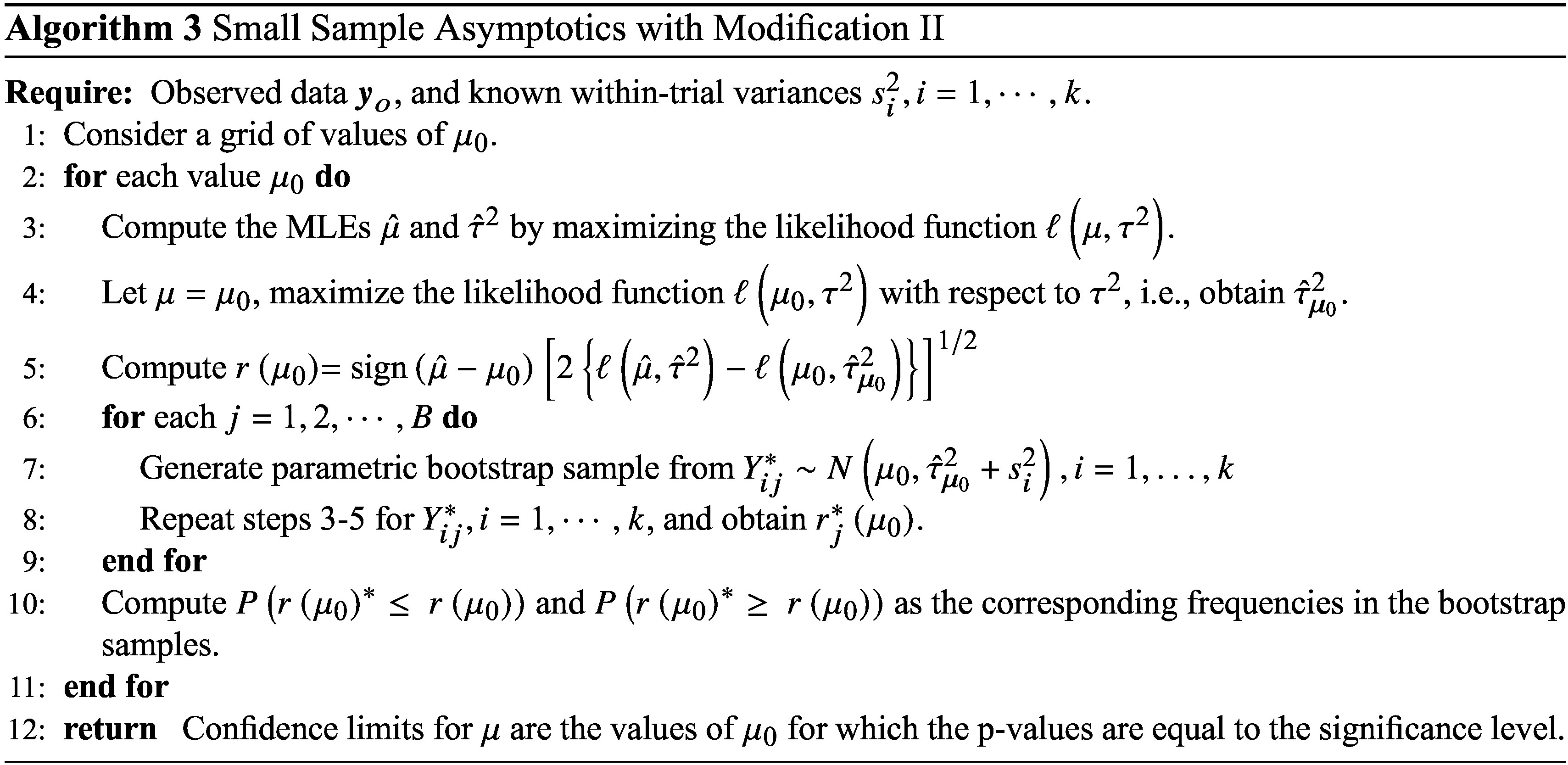

For the numerical implementation of the proposed methods, a grid of values for 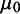 can be considered for both algorithms. The grid of values 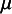 can be selected to cover a range around the MLE of 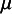 , since the upper confidence limit will be larger than the MLE of 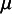 , and the lower confidence limit will be smaller. For the algorithm for modification I, to calculate a two-sided confidence interval for 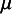 , the upper and lower confidence limits are obtained by finding the values of 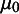 for which 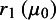 is close to the standard normal quantiles according to a specified threshold. For the algorithm for modification II, the confidence limits for 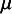 are determined by finding the values of 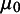 that make the *p* values 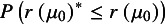 and 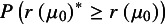 close to the significance level, once again according to a specified threshold.

## 3 Results

### 3.1 Simulation settings

In order to evaluate the performance of the proposed methods, a simulation study similar to that of Rover et al.^1^ was conducted for the modified HKSJ (mKH) method with a small number of studies (*k*). The “relative amount of heterogeneity” is expressed in terms of the measure 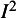 defined as (20)

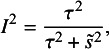

where 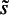 is an “average” standard error among the study-specific 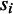 values.^1^^,^ ^14^ In the simulation study, 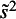 was calculated as the arithmetic mean of the squared standard errors, i.e., (21)

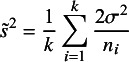

with 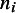 being the group size of trial 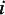 ( 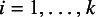 ) and 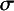 is a scaling factor. Since the results depend solely on the ratio 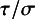 , 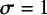 was set in the simulations.^15^ The simulation setup we have used and also adopted in the work of Rover et al.,^1^ is taken from the study by IntHout et al.^15^

Similarly to the work by Rover et al.,^1^ four different meta-analyses scenarios were considered in the simulation: (A) trials of equal size, (B) equally sized trials but including one small trial, (C) with half large and half small trials, and (D) equally sized trials and one large trial. A large trial is 10 times the size of a small trial, which means that the associated standard errors differ by approximately a factor of 3.^1^ The number of trials *k* ranged from 2 to 10, that is, 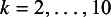 . The true level of heterogeneity was set at 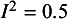 ; see (20). The average group size 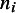 in each trial was set at around 100. Without loss of generality, the true value of 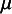 was set to zero. The 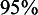 confidence interval (CI) of 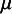 was constructed using the proposed methods and compared to the HKSJ method^3^ and the modified HKSJ (mKH) method for small *k*.^1^

The simulation for normally distributed outcomes under the model (3) involved several key steps, as detailed in Appendix 2 of the work of IntHout et al.^15^ For each scenario with 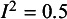 , the variance 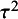 was calculated using equations (20) and (21). For each trial 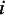 , the ‘true’ trial-specific effect size 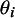 in (2) was drawn from a normal distribution with mean 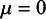 and variance 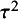 . The trial outcomes were then generated from a normal distribution with mean 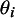 and variance 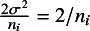 (recall that the scaling factor 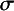 is chosen to be equal to one). Additionally, for each trial *i* with group size 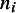 , the variance of the trial outcome, i.e., the quantity 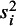 , was generated based on a 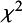 distribution with 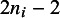 degrees of freedom, divided by 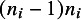 ; see the work of IntHout et al.^15^ For each simulation, the generated observed trial outcomes 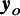 and within-trial variances 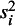 for 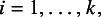 were used as input for the proposed algorithms to construct 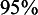 CIs of 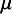 . For the fiducial approach, 5,000 realizations of the fiducial quantities were used to obtain the confidence limits. For asymptotic modifications I & II, 1000 bootstrap samples were used, i.e., 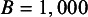 .

**Figure 1:**
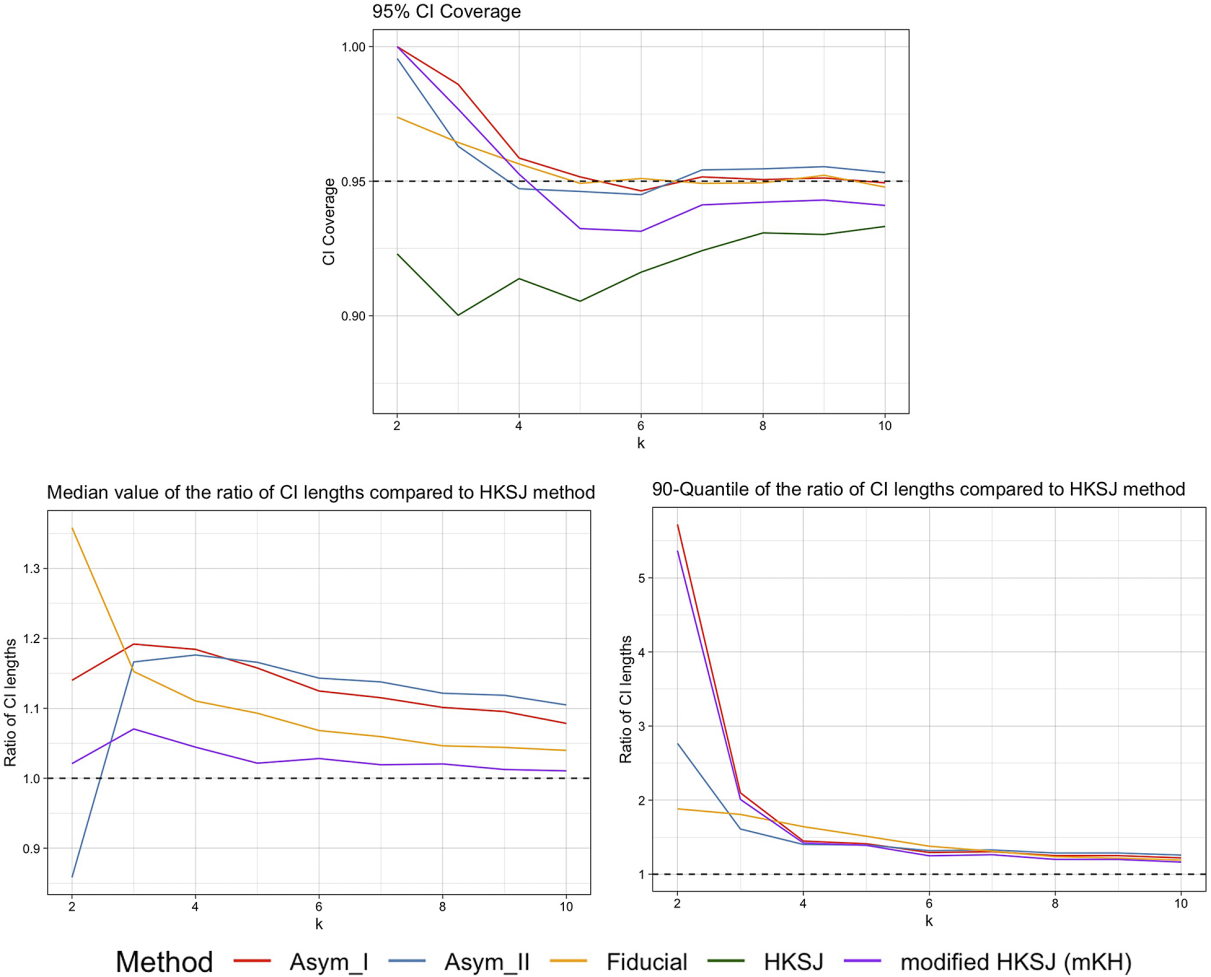
*Plots of the 95% CI coverage, median and 90th quantiles of the CI width ratios compared to the HKSJ method for trials with half large/small sizes and average size around 100 (with a ceiling* ( 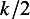 ) *of small sizes for kodd), by Asymptotic Modification I (Asym_I) method, Asymptotic Modification II (Asym_II) method, Fiducial approach, HKSJ method, and modified HKSJ (mKH) method.*

**Figure 2:**
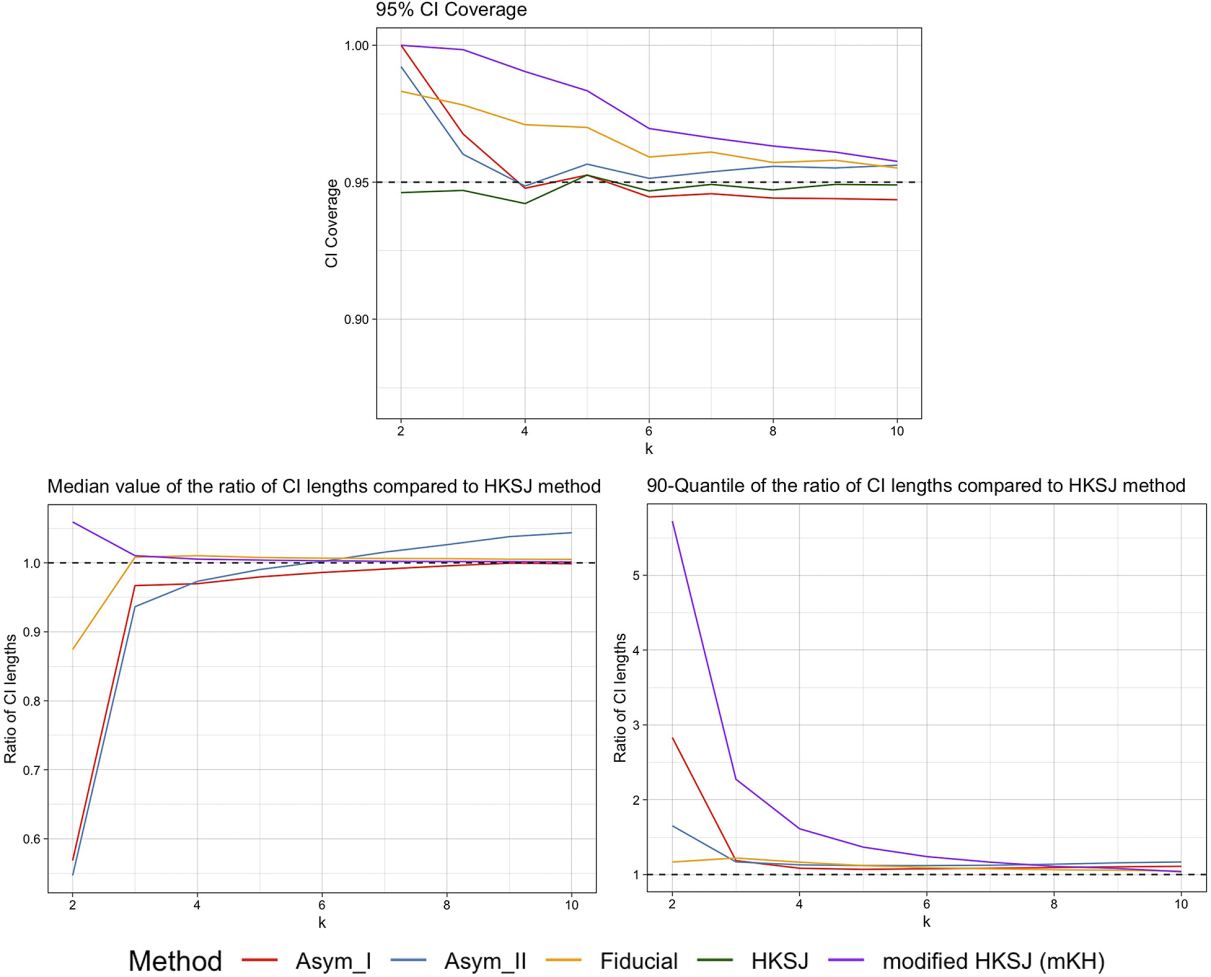
*Plots of the 95% CI coverage, median and 90th quantiles of the CI width ratios compared to the HKSJ method for trials with equal sizes of* 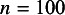 , *by Asymptotic Modification I (Asym_I) method, Asymptotic Modification II (Asym_II) method, Fiducial approach, HKSJ method, and modified HKSJ (mKH) method.*

**Figure 3:**
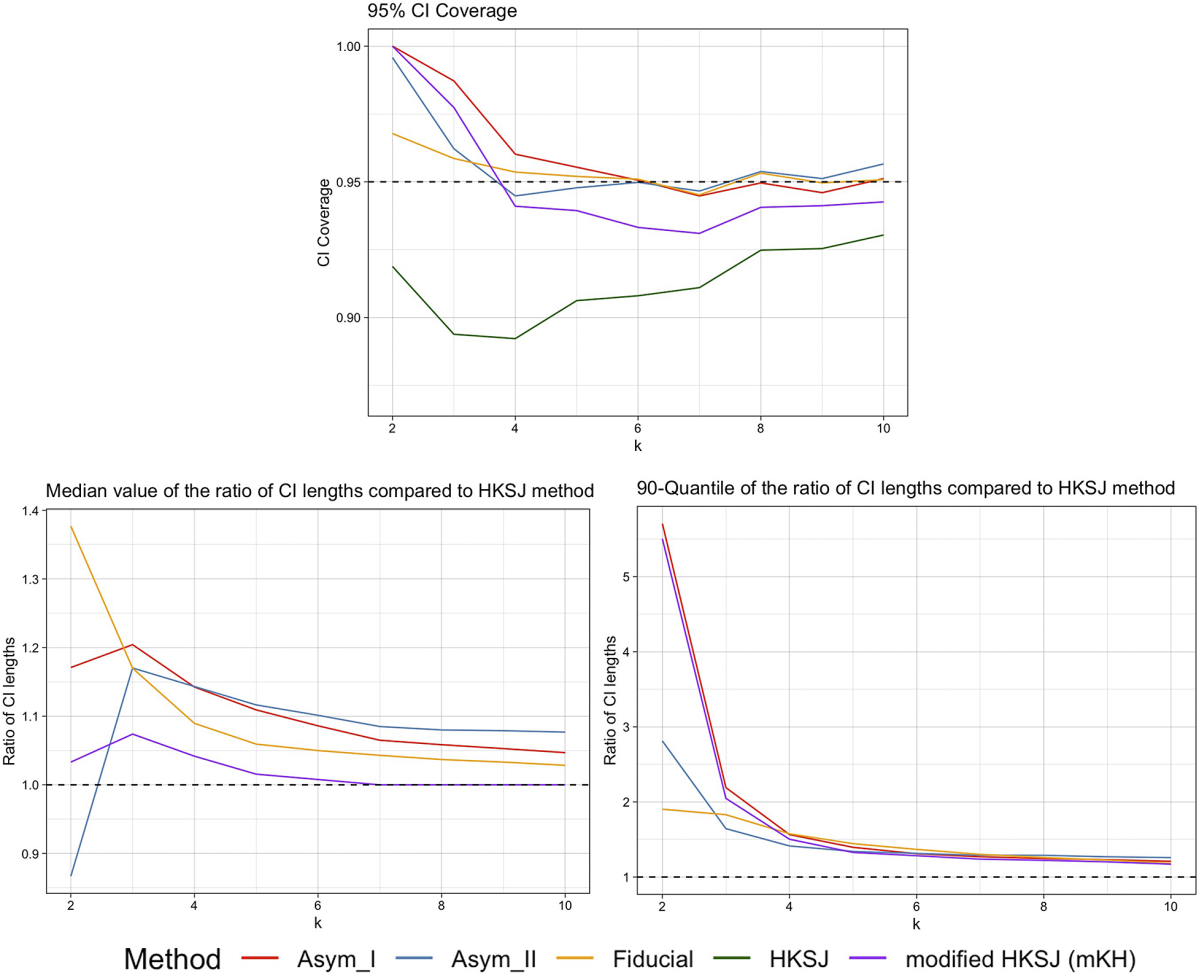
Plots of the 95% CI coverage, median and 90th quantiles of the CI width ratios compared to the HKSJ method for the case of equally-sized trials with one large trial and average size around 100, by Asymptotic Modification I (Asym_I) method, Asymptotic Modification II (Asym_II) method, Fiducial approach, HKSJ method, and modified HKSJ (mKH) method.

**Figure 4:**
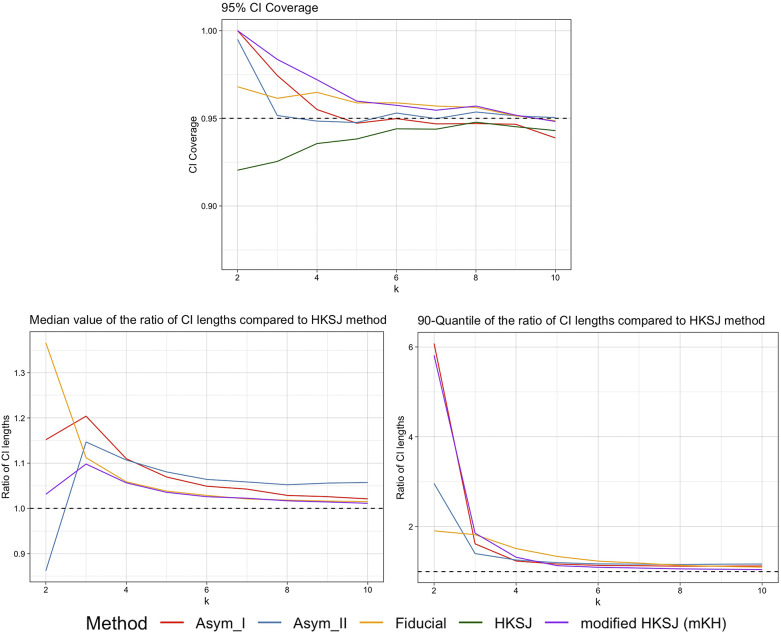
Plots of the 95% CI coverage, median and 90th quantiles of the CI width ratios compared to the HKSJ method for the case of equally-sized trials with one small trial and average size around 100, by Asymptotic Modification I (Asym_I) method, Asymptotic Modification II (Asym_II) method, Fiducial approach, HKSJ method, and modified HKSJ (mKH) method.

To compare the proposed methods with the existing ones, the CIs of 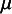 were also constructed using the HKSJ method^3^ and the modified HKSJ (mKH) method.^1^ The DerSimonian–Laird (DL) estimator^16^ of the between-study variance 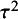 was used for both the HKSJ and mKH methods in the simulation. According to Rover et al.^1^ and Sánchez-Meca and Marín-Martínez,^17^ the constructed CIs for both the HKSJ and mKH methods are similar if using the DL estimator, the restricted maximum likelihood (REML) estimator,^18^ and the Paule–Mandel (PM) estimator.^12^

A total of 5,000 meta-analyses were simulated, and all simulations were performed using R.^19^ In order to compare the different approaches for computing the confidence intervals for 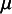 , the coverage probabilities and the expected widths of the CIs were calculated, where the latter was expressed as a ratio to the expected length of the interval obtained by the HKSJ method. The median of these ratios and the 90th percentile of the ratios were also evaluated, as in the work of Rover et al.^1^

### 3.2 Simulation results

For trials with half large and half small sizes, with the average size around 100, the simulation results are shown in Figure 1. The results include 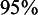 CI coverage, median and 90th quantiles of the expected CI length ratios mentioned above, for the number of trials 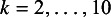 . A large trial was 10 times the size of a small trial, with the overall trial average being around 100, and the number of small-sized trials was chosen to be 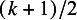 or 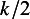 depending on whether *k* is odd or even. For example, for 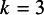 , the group sizes for the trials would be 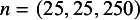 ; for 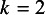 , the group sizes would be 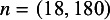 .

The simulation results demonstrate that the proposed new methods, including the fiducial approach and the small sample asymptotic methods, particularly Asymptotic Modification II, perform better than the modified HKSJ (mKH) method when the number of trials is small. The HKSJ method underperforms for small 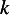 , and while its performance is indeed enhanced by the modified HKSJ (mKH) method, the proposed new methods offer superior results. The fiducial approach consistently provides more accurate confidence interval (CI) coverage. Asymptotic Modification II, in particular, shows more accurate CI coverage and shorter CI lengths for small numbers of trials ( 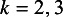 ), compared to other methods. Additionally, Asymptotic Modification I also offers a more accurate CI coverage when 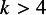 . The fiducial approach yields larger median CI lengths, but with less variability in CI lengths, especially for 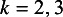 . On the other hand, Asymptotic Modification I results in CI lengths similar to mKH but with a larger median, and Asymptotic Modification II shows smaller CI lengths for 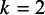 and less variability of CI lengths for 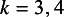 .

Figure 2 displays the simulation results for trials with equal group sizes of 100. In this scenario, the HKSJ method can handle small *k* effectively. It works very well when the analyzed studies are of equal size (i.e., have equal standard errors), as shown in the work of Rover et al.^1^

Figures 3 and 4 show the corresponding simulation results for the scenarios with one large trial, and with one small trial, respectively, with an average group size around 100. The results and conclusions are similar as in the case of trials with half-large and half-small sizes. The HKSJ method underperforms for small 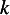 , while the mKH method improves its performance. The fiducial approach consistently provides more accurate CI coverage close to the nominal 95%. Asymptotic Modification II maintains more accurate CI coverage, outperforming the mKH method for small numbers of trials. For the CI lengths, the results reveal that the fiducial approach yields larger median CI lengths with less variability, while Asymptotic Modification II shows smaller and more stable CI lengths for small 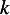 , indicating its robustness.

In summary, for computing a CI of 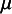 in unbalanced settings with a small number of studies *k*, Asymptotic Modification I shows results comparable to those based on existing methods. However, the fiducial approach and Asymptotic Modification II show better results, with more accurate CI coverages and shorter CI lengths. The findings highlight the superior performance of the newly proposed methods in terms of both CI coverage and length, making them excellent choices for practical applications.

## 4 Examples

An alternative methodology, not focused on in our work, is a Bayesian approach (see the works of Bender et al.,^20^ and Friede et al.,^21^) which introduces stability through the incorporation of prior knowledge about the heterogeneity parameter 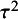 through (weakly) informative prior distributions. The two examples of few studies illustrated in the work of Bender et al.^20^ are used here to demonstrate the performance of the proposed methods. These examples come from dossier assessments conducted by the Institute for Quality and Efficiency in Health Care.^20^ Assuming that the logarithm of hazard ratios (HR) or relative risks (RR) follows a normal distribution, 95% confidence intervals for the data in both examples were generated using the HKSJ method, the modified HKSJ (mKH) method, the Bayesian approach, and the proposed methods, including the Fiducial approach and Asymptotic Modification I and II. For Bayesian approaches, half-normal priors for the heterogeneity parameter 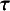 with scales of 0.5 (Bayesian-HN(0.5)) and 1.0 (Bayesian-HN(1)) were applied.

**Figure 5:**
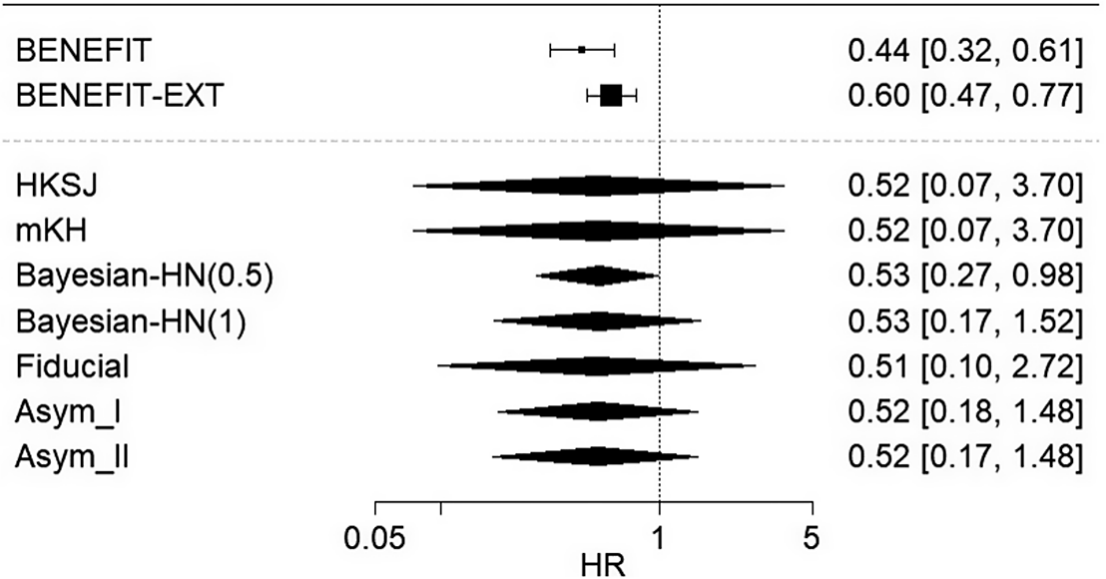
*A forest plot showing random-effects meta-analysis results for the first belatacept example, by HKSJ method, modified HKSJ (mKH) method, Bayesian method using half-normal priors for* 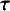 *with scales 0.5 (Bayesian-HN(0.5)) and 1 (Bayesian-HN(1)), Fiducial approach, and Asymptotic Modification I (Asym_I) and II (Asym_II) methods.*

In the first example, the added benefit of belatacept compared to ciclosporin A in combination with corticosteroids and mycophenolate mofetil was assessed as the appropriate comparator therapy for the prophylaxis of graft rejection in adults receiving a renal transplant.^20^ Only 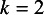 studies were available. The results for this example are illustrated in Figure 5. The HKSJ method yielded a wide 95% CI due to insufficient data to reliably estimate heterogeneity, leading to a conservative result. The mKH method produced the same result as HKSJ in this case. The Bayesian approach provides an alternative, yielding narrower intervals than HKSJ and mKH. However, prespecification of the prior distribution for between-study variation 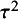 is crucial to interpreting the intervals in a frequentist sense, as a hypothesis test.^20^ The application of a half-normal prior with scale 0.5 for 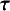 results in a statistically significant pooled effect estimate, while a scale of 1 does not. Among the proposed methods, Asymptotic Modifications I and II generate similar but slightly shorter confidence intervals compared to the Bayesian method with a half-normal prior of scale 1. The Fiducial approach provides slightly wider intervals than the asymptotic modification methods but remains narrower than those from HKSJ and mKH.

**Figure 6:**
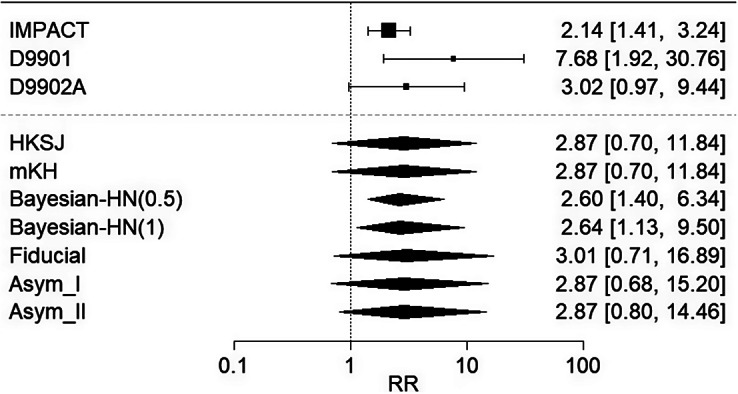
*A forest plot showing random-effects meta-analysis results for the second sipuleucel-T example, by HKSJ method, modified HKSJ (mKH) method, Bayesian method using half-normal priors for* 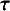 *with scales 0.5 (Bayesian-HN(0.5)) and 1 (Bayesian-HN(1)), Fiducial approach, and Asymptotic Modification I (Asym_I) and II (Asym_II) methods.*

In the second example, the added benefit of sipuleucel-T compared to the appropriate comparator therapy to treat asymptomatic or minimally symptomatic metastatic (non-visceral) castrate-resistant prostate cancer was assessed in male adults for whom chemotherapy is not yet clinically indicated.^20^ Here, 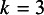 relevant studies were available. The results for this example are shown in Figure 6. Bayesian approaches yield shorter confidence intervals, indicating a statistically significant difference between treatment groups, to the disadvantage of sipuleucel-T. The mKH method produces the same wide confidence interval as HKSJ, including the relative risk of 1. The three proposed methods generate wider confidence intervals, particularly on the right-hand side, covering the finding from the second study with a large upper confidence limit for the relative risk, which may suggest better coverage of the true value. Although Bayesian approaches tended to give qualitatively different results relative to the other methods, the choice of method generally had negligible impact on the conclusions drawn in this example.

## 5 Discussion

In this work, we have proposed two novel methods for computing confidence limits for 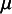 in random-effects meta-analysis, particularly suited for a small number of studies. One method is based on fiducial inference, while the other involves two small sample asymptotic procedures, referred to as Asymptotic Modifications I and II. Our simulation studies indicate that for unbalanced settings, when the number of studies *k* is small, the proposed methods show significant improvements compared to existing methods. The fiducial approach and Asymptotic Modification II exhibit superior performance, providing more accurate CI coverages and shorter CI lengths compared to those based on the modified HKSJ method proposed by Rover et al.^1^ Also, Asymptotic Modification I provides results that are comparable to those based on the modified HKSJ method. We believe that these findings have important practical implications since meta-analyses with a small number of studies are frequently encountered in applications. It is noted that for 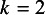 , none of the competing approaches yields useful information, so the differences in relative performance of the approaches do not have practical relevance.

For methods based on small sample asymptotics, other modifications of the statistic 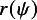 given in (18) are also available in the literature, with the goal of achieving improved small sample performance; see the work of Brazzale et al.^13^ Such modifications have in fact been applied in the context of the model and problem investigated in our work; see the work of Guolo.^22^ Figures 1-3 in the work of Guolo^22^ show that such modifications exhibit a performance quite similar to what we have noted for Asymptotic Modifications I and II considered in our investigation. We also want to point out that in the context of inter-laboratory studies where the model (3) is assumed, but with unknown within-trial variances, the fiducial approach was developed in the work of Tian^23^ and small sample asymptotic procedures were investigated by Sharma and Mathew.^24^ Furthermore, Zejnullahi and Hedges ^25^ introduced two alternative robust variance estimators were introduced by Zejnullahi and Hedges^25^ for small meta-analyses, evaluating degrees-of-freedom adjustments for confidence intervals.

In addition to the methodological contributions, we are developing an R package that will offer a practical tool for the easy implementation of the proposed methods.

Future research could explore the impact of estimated within-trial variability on the performance of the CIs of 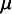 . Furthermore, the median or mean of the fiducial quantities could be used as a point estimate of 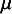 . The performance of such a point estimate of 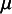 could be evaluated and compared with the maximum likelihood estimate (MLE), the restricted MLE (REML), and the estimate resulting from the DerSimonian–Laird (DL) approach. These are currently under investigation.

## Supporting information

Duan et al. supplementary material Duan et al. supplementary material

## Data Availability

Program codes, simulated data summary, and real data examples supporting the findings of this study can be found in the Supplementary Material.

## Supplementary material

The supplementary material for this article can be found at http://doi.org/10.1017/rsm.2025.10022.
